# Changes in Muscular Activity in Different Stable and Unstable Conditions on Aquatic Platforms

**DOI:** 10.3390/biology11111643

**Published:** 2022-11-10

**Authors:** Ana Conceição, Orlando Fernandes, Miguel Baia, Jose A. Parraca, Bruno Gonçalves, Nuno Batalha

**Affiliations:** 1 Department of Sport Sciences, Sport Sciences School of Rio Maior, 2040-413 Rio Maior, Portugal; 2 Research Centre in Sports, Health and Human Development (CIDESD), 5000-801 Vila Real, Portugal; 3 Departamento de Desporto e Saúde, Escola de Saúde e Desenvolvimento Humano, Universidade de Évora, 7000-654 Évora, Portugal; 4 Comprehensive Health Research Centre (CHRC), University of Évora, 7004-516 Évora, Portugal

**Keywords:** squat exercises, plank exercises, electromyography, aquatic platforms

## Abstract

**Simple Summary:**

Use of aquatic platforms in swimming pools is growing through their utilisation by individuals and group classes as a tool for promoting instability through water turbulence. Little is known about variations in muscle recruitment due to the introduction of instability, and the differences between types of exercise performed in a land (stable) environment and then performed on an aquatic platform (unstable). This study attempted to understand the changes in muscular activity of the erector spinae, biceps femoris, rectus femoris, external oblique, and rectus abdominis during the squat and plank in stable and unstable environments. For this purpose, participants performed squat and plank exercises in a stable environment (on land) and an unstable environment (on an aquatic platform) for 10 s with 40 s of rest. The results provide excellent evidence of muscular recruitment during squat and plank exercises, suggesting that exercises in unstable conditions on an aquatic platform could be a training alternative since water turbulence causes a slight increase in muscle activation.

**Abstract:**

The present study aimed to analyse and compare the muscle activity of twelve participants (seven men and five women) (age 20.1 ± 0.9 years; height 170.5 ± 10 cm; body mass: 64.86 ± 8.3 kg) in two exercises, each with two variants: squat (dynamic and static) and plank (hands and elbows) in a stable environment on land and an unstable environment on an aquatic platform. The erector spinae, biceps femoris, rectus femoris, external oblique, and rectus abdominis muscles were evaluated using surface electromyography. The dynamic squat increases the recruitment of the biceps femoris and external oblique, while the static squat demands greater activation of the rectus femoris. The elbow plank exercise increases the recruitment of erector spinae muscles, and the hand plank exercise increases the recruitment of the erector spinae and external oblique. In conclusion, performing exercises in unstable conditions on an aquatic platform slightly increases muscle recruitment.

## 1. Introduction

Squat and plank exercises are essential in a training programme because they provide benefits by stimulating the lower limb and core muscles, and the importance of strengthening and resistance of those muscles has been reported to prevent sports injuries [1].

The squat exercise has a long history in fitness training, exercise for rehabilitation, and strength training for performance in sport [2]. It is a functional movement performed loaded or unloaded by flexing and extending the hip, knee, and ankle joints. The squat exercise is regarded as a closed kinetic chain exercise, where the force is expressed through the end (length) of the limb while it is fixed to the ground [2,3,4]. The plank, like the squat, is an exercise performed with only the body weight that can be executed dynamically or statically, with hands or elbows resting on the ground [5].

Kinematic, kinetic, and electromyographic (EMG) studies have reported differences in lower limb muscle activation resulting from variations in squat depth, foot placement, training status, and training intensity [6], for instance, in unilateral and bilateral squatting exercises such as single- [7] or double-leg squats and lunges performed on stable and unstable surfaces [8,9]. 

Although the squat is a widely accepted exercise to strengthen the thigh musculature, it presents variations due to the stance width and depth of the squat [3]. However, no significant difference in quadriceps muscle activation patterns has been noted when comparing narrow and wide stances and varying foot positions [10].

A poorly performed squat may result in altered lower extremity alignment, exposing the lower extremity joints to excessive torque that may require adaptive muscle activation strategies to stabilize them [10]. The squat consists of two phases: descending and ascending, each corresponding, respectively, to the eccentric and concentric phases of the movement [11].

Due to the synergistic muscle actions involving a coordinated contraction of the hamstrings and quadriceps, several squat exercises using different levels of stability (a double- or single-leg squat on stable or unstable surfaces) have been proposed to enhance knee stabilization and potentially avoid excessive valgus and varus in athletes [2]. Performing the exercise on different surfaces and using a combination of different squatting movements have been suggested as practical strategies to improve neuromuscular control and prevent injuries in athletes [12]. The muscles involved are the quadriceps, namely the rectus femoris, vastus lateralis, and vastus medialis; the hamstrings (biceps femoris and semitendinosus); and the erector spinae [13].

During recent years, suspended exercise modalities have emerged as a highly effective way of increasing trunk activity through unstable exercises. For instance, the suspended version of push-ups shows superior lumbar multifidus activity to either the stable version or the exercise performed with other unstable bases [14]; in addition, push-ups performed with dual instability have significant muscular activity compared to single instability or stable push-ups [15]. In the same way, suspended front planks have demonstrated higher rectus abdominis [14,16] and lumbar erector spinae [16] activity than the stable version.

According to Ni et al. [17], in a normal plank exercise, the most-recruited muscles are the external oblique, erector spinae, longissimus thoracis, gluteus maximus, and rectus abdominis. Plank exercises are a method used to work core muscles and are one exercise used to improve core stability [18]. Planking activates the core muscles, sparing high compressive forces on the lumbar vertebrae [19]. So, it is important to activate these muscles, mainly the abdominals, to keep the spine neutral [16].

Exercises performed on unstable platforms create oscillations and faster motor responses, improving muscle reaction [20]. Although bringing undeniable advantages in specific situations, there is no consensus in the scientific community that the practice of exercising in unstable environments is always more efficient and desirable than performing the same exercises in a stable environment [21].

One trunk muscle-training method that has received considerable attention is the use of instability, a common resistance training method used in exercise programmes. This can be obtained through the use of many devices and techniques, including, but not limited to, unstable platforms such as BOSU balls or Swiss balls, and/or completing open kinetic chain exercises with free weights [22]. Instability tends to trigger involuntary dynamic motor reactive responses in order to generate better neuromuscular control, especially in the joints [23].

When comparing core muscle activity during plank exercises, Mok et al. [24] found that the EMG activity was higher in the transversus abdominis and internal abdominal obliques when using a suspension device (TRX^®^ PRO Suspension Trainer, Fitness Anywhere LLC, San Francisco, CA, USA). Besides suspension devices, exercises on water are also becoming an ideal environment to correct neuromuscular communication, along with helping balance and movement coordination [25].

Instability devices have become very popular in different types of training; this instability can be achieved by using unstable surfaces instead of stable surfaces. Aquatic platforms are growing in popularity through their use in individual or group classes in swimming pools as a tool to promote instability through water turbulence. 

However, little is known about the muscle activation patterns in unstable exercises on aquatic platforms and the differences between those and patterns in stable conditions on land during exercises such as squats and planks that can be used in classes. 

To the best of our knowledge, no studies have investigated muscular activity in stable conditions and on unstable aquatic platforms for squats and planks. Such a study will provide useful information for the proper integration of different squat and plank exercises in class programmes. Therefore, this study aimed to analyse and compare muscular activity of the erector spinae (ES), biceps femoris (BF), rectus femoris (RF), external oblique (EO), and rectus abdominis (RA) during the squat and plank in stable and unstable environments (aquatic platform).

We hypothesize that performance of squat and plank exercises will have greater EMG activity on an unstable aquatic platform compared with the stable environment.

### 1.1. Participants

Twelve participants participated in the study (age 20.1 ± 0.9 years; height 170.5 ± 10 cm; body mass: 64.86 ± 8.3 kg; mean ± SD), seven male and five female, with inclusion criteria of each participant presenting low adiposity (determined by impedance levels around 5 and 50 kΩ) and already having had some experience with aquatic platforms. Everyone volunteered to participate in this study and provided a written informed consent. This research was approved by the ethics committee of the funding institution. The research was undertaken in compliance with the Declaration of Helsinki and the international principles governing research on humans and animals.

### 1.2. Data Collection 

The experiments were performed in an outside environment near a swimming pool. Subjects performed a general standard warm-up that consisted of a 10 min warm-up performed on a treadmill at an intensity corresponding to a rating of perceived exertion between 8 and 10 on the Borg scale range. After that, they performed two exercises with two variants each: (i) squat (dynamic and isometric) and (ii) plank (hands and elbows) for 10 s each with an interval of 40 s, in stable and unstable conditions (Table 1).

**Figure 1:**
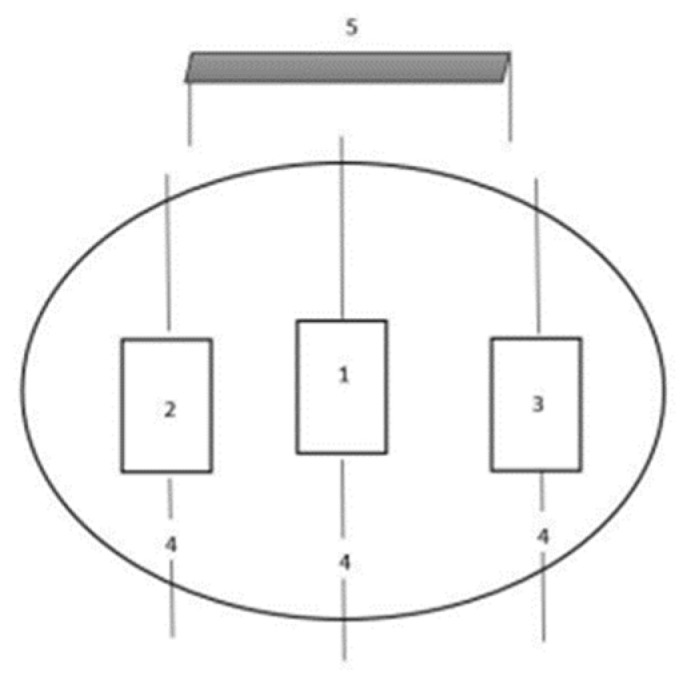
Representative scheme. 1—Test platform where each subject performed data collection; 2—Platform for creating turbulence on one side (subject’s right side) and on both sides; 3—Platform for creating turbulence on both sides (subject’s left side); 4—Ropes with moorings to the platform and out of the water; 5—Data collection table to receive the EMG data.

The stable conditions were on the floor near the pool, and the unstable conditions were on an aquatic platform (Fitness on Water, Hydrorider, Portugal; 200 cm × 80 cm × 10 cm; 178 cm × 76 cm). The aquatic platform was positioned in the middle of the swimming pool where the subjects were tested and lashed in four places to maintain a static position. Turbulence was created by two participants on the left and right side (Figure 1) of each subject evaluated. The participants responsible for simulating the turbulence for both sides were instructed to balance their feet and legs in the bipedal position, in order to move the aquatic platform and create the respective turbulence in the water. Surface EMG signals from the ES, BF, RF, EO, and RA muscles on the right side of the body were measured. These muscles were selected according to their importance in the squat [11] and plank exercises [14].

Bipolar surface electrodes were used (10 mm diameter discs; Plux, Lisbon, Portugal) with an inter-electrode distance of 20 mm. Electrodes were placed in the middle of the line that connects the acromion process and the manubrium (sternum), two fingers below the clavicle [26]. The electrodes were placed on ES, BF, RF, EO, and RA muscles as per SENIAM recommendations [27]. The skin under the electrodes was shaved, rubbed with sandpaper, and cleaned with alcohol so that the inter-electrode resistance did not exceed 5 kΩ [28]. The ground electrode was positioned over the cervical vertebrae. Transparent dressings with labels (10 cm × 12.5 cm; Hydrofilm^®^, by HARTMANN USA, Inc. 481 Lakeshore Parkway, Rock Hill, SC, USA) were used to cover the electrodes to isolate them from the water [29]. The EMG equipment, composed of a wireless EMG device (BioPLUX.research, Lisbon, Portugal) with a sampling rate of 1 kHz, was fixed in a waterproof bag and placed in a backpack on each subject, and the data were transmitted to the PC in real time. Once all electrodes were placed, maximal dynamic contractions (MDC) were measured to normalize all EMG signals [30,31]. Once all EMG data were normalized, subjects performed isometric and dynamic squats and elbow and hand planks in stable and unstable conditions. The exercises were performed in a randomized order to prevent data fatigue errors. A certified strength and conditioning specialist (NSCA-CSCS) instructed all subjects about the proper technique. If any subject was not able to maintain proper technique as instructed, then all data were omitted from the analysis process.

### 1.3. Exercise Trials

Isometric Squat: Thighs should be parallel to the floor and knees bent at an angle of 90° [32], without the back and head supported, which makes it more difficult since it is important to keep the back and head straight during the movement so that it does not put pressure on the lumbar and cervical areas [32].

Dynamic Squat: The participant starts standing with an anatomically correct posture and with feet shoulder width apart, then they must squat down to 90° flexion of the knee without it passing the tip of the foot [33].

Elbow Plank: The elbow plank is performed with the elbows flexed at 90°. During the exercise, the torso should be straight, and the legs, head, pelvis, and spine should be neutral [14].

Hand Plank: This is performed with the hands resting on the floor at shoulder width. The arms and legs are in extension and the hips are in a straight line with the rest of the spine, keeping the abdominal muscles contracted [5].

### 1.4. Data Analysis

This study was based on a process of automatic analysis. Therefore, all EMG analysis was carried out without any manual intervention, using only automatic instruments developed by MATLAB. EMG analyses were performed with a MATLAB routine starting with a raw signal, then DC components were removed and filtered with a fifth-order Butterworth bandpass, where the lower and upper cut-off frequencies were set to 10 and 500 Hz, respectively. The EMG magnitude (%) of each active phase was estimated and plotted as a function of time.

Data processing started out with descriptive analysis (mean + standard deviation) for all the results obtained for each exercise. Assumptions of statistical tests such as normal distribution (Shapiro–Wilk test, *p* > 0.05) and sphericity (Mauchly test, *p* > 0.05) of data were checked as appropriate. All the parameters were normally distributed. Then the parametric statistical technique was performed using the repeated measures test (ANOVA). If there were significant differences (*p* value < 0.05), post-hoc and magnitude tests were performed to identify the context of those differences between exercises.

## 2. Results

Table 2 shows that elbow planks and hand planks presented significant differences in relation to ES muscle activation (*p* = 0.015; η²*p* = 0.268 and *p* = 0.009; η²*p* = 0.29, respectively). The post-hoc test revealed that elbow plank exercises showed the most significant differences in ES muscle activation between land and aquatic execution with turbulence on one side, and it also occurred between land and aquatic execution with turbulence on both sides. On the other hand, for the hand plank exercises, a significant difference in muscle activation occurred between land and aquatic exercises with turbulence on both sides.

Regarding activation of the RF muscle depending on context, we can verify through Table 3 that the only exercise that showed a significant difference was the isometric squat (*p* = 0.034; η²*p* = 0.227). In order to analyse which contexts of the isometric squat led to a significant difference in muscle activation, the post-hoc difference was determined in pairs tests. It was determined that the differences in muscle recruitment were significant for the isometric squat exercise only in the change of context between land and the aquatic environment with turbulence on both sides, with muscle recruitment being superior at the medium level of unstable conditions.

Table 4 shows us that, regarding the activation of the BF muscle, the only exercise where significant differences were verified was the dynamic squat (*p* = 0.013; η²*p* = 0.277). Significant differences were revealed between land and aquatic exercises with turbulence on one side. It was also possible to identify differences between the land environment and the aquatic environment with turbulence on both sides, with an increase in recruitment of the BF muscle because of instability.

For the RA muscle, there were no significant differences between the performance of the planking and squatting exercises performed in an unstable aquatic environment or on land (Table 5).

Lastly, in Table 6, we found that the dynamic squat and hand plank exercises showed a quite significant difference in activation of the EO muscle regarding the context (*p* = 0.003; η²*p* = 0.336 and *p* = 0.008; η²*p* = 0.296). The post-hoc differences in the pairs test showed statistically significant muscle activation for the dynamic squat between performance on land and on an aquatic platform with turbulence on both sides, and between aquatic exercise without turbulence and aquatic exercise with turbulence on both sides. It should also be noted that the hand plank showed significant differences between land exercise and aquatic exercise without turbulence as well as aquatic exercise with turbulence on both sides.

## 3. Discussion

The purpose of the present study was to analyse muscle recruitment in squat and plank exercises performed in stable and unstable conditions. We hypothesized that performing the exercises in unstable conditions would increase muscle activity compared to stable conditions. Therefore, the initial hypotheses were partially supported.

The primary finding of this study was that changes in the recruitment of specific muscles during squat and plank exercises appear to be elicited when performed on an aquatic platform. Based on EMG activations, our study demonstrated increased ES and EO activation when planks were performed on the aquatic platform with turbulence compared with the plank performed on land; increased BF and EO activation in the dynamic squat was observed, as well as RF activation in the isometric squat performed on an aquatic platform. Those exercises can easily be modified to increase muscle activity and level of difficulty by creating oscillations and faster motor responses through unstable devices or conditions [34].

Since there was no evidence of muscle activation while performing exercises on aquatic platforms, this study provides a comparison of two types of exercise performed in a land (stable) environment and then performed on an aquatic platform, to identify possible variations in muscle recruitment due to the instability factor.

The results of this study are consistent with those of previous studies indicating that instability devices (for example the Swiss ball) elicit greater EMG activity of the EO muscle compared with traditional exercises designed to target the abdominal wall [31]. Trunk musculature is vital for ensuring integrity of the vertebral column and resisting excessive rotation during isometric contractions [35]. Therefore, the increased EMG activity of the RA and EO when planks are performed with an instability device, for example the TRX or Swiss ball, may also be caused by a greater demand for core stabilization to prevent or resist spinal perturbations during the exercise [35].

In terms of squat exercises, it is known that the muscles involved are the quadriceps (RF, vastus lateralis, and vastus medialis), the hamstrings (BF and semitendinosus), and the ES [13] when performed in a stable condition. The squat is a widely accepted exercise to strengthen the thigh musculature when performing exercises in stable conditions [3]. The concept of squat stabilization has been investigated in terms of stable vs. unstable surfaces. Saeterbakken et al. [36] found that there was greater muscle activity in the abdominal stabilizer muscles when the exercise was performed on an unstable surface (balance disks) when compared to a stable surface (Smith machine squat). When squatting on unstable compared to stable surfaces, it is possible that the trunk, instead of the lower limb muscles, works as the primary stabilizer to maintain balance [11]. The results of the present study agree with these statements, showing that there are significant differences in the isometric squat exercise when performed on an aquatic platform, and concluding that this exercise, in a context of instability, significantly influences the degree of recruitment of the RF muscle.

However, when performing dynamic squat exercises, the muscles that showed more activation were the BF and EO. According to Monajati et al. [11], the greater activation of both the medial hamstring and quadriceps during a single-leg squat on a BOSU suggests that performing this exercise may be a better option compared to the double-leg squat on a BOSU balance trainer to decrease the risk of injuries.

Another interesting finding during this study was that the RA muscle showed no significant differences in activation. However, this last result goes against the unanimity of other investigations such as those of Snarr et al. [14] and Lehman, Hoda, and Oliver [37], since they showed that abdominal muscle groups are demanded in the performance of several planking exercises in an unstable context. This might confirm that there are significant differences between instability devices, as shown by Snarr and Esco [14].

Furthermore, it was shown that in plank exercises, when the condition changed from stable to unstable, there was an increase in ES muscle recruitment if the individual suffered turbulence on both sides. Therefore, we can conclude that muscle recruitment is significantly higher in elbow and hand plank exercises when performed in an aquatic environment with turbulence on both sides. There is in fact a progressive increase in activation of the muscle due to an increase in turbulence in the aquatic environment. On the other hand, the obtained results suggest that performing the dynamic squat exercise increases muscle activation (BF and EO), namely when the exercise is performed in an aquatic environment with turbulence on both sides. In this way, muscles present a higher degree of activation when there is a greater turbulence in the aquatic environment.

Since it seems that using aquatic platforms can increase muscular activity in the muscles studied, perhaps the aquatic platform could be incorporated in training programmes for those wanting increased muscle activation during plank and squat exercises. Likewise, performing exercises with instability appears to elicit an intense response and an adequate muscle stimulus during certain exercises.

This study has several limitations. All the participants were between the age of 19 and 20 years old, so the interpretation of the results does not apply to those over 20 years old or elderly populations. There are other limitations related to the methodology, which were: (i) the small number of muscles analysed with EMG, because of the limitation in the number of channels of the device used in this study; (ii) we did not analyse the contribution of muscle agonists, antagonists, or synergists in the different exercises; (iii) the protocol only had two participants simulate the turbulence in the water for the subject evaluated, instead of making the protocol reflect group class context.

## 4. Conclusions

The exercises performed on an aquatic platform elicited high-level muscle recruitment during squat and plank exercises. Therefore, we have shown significant increases for different muscles in exercises performed in an unstable environment, namely in the dynamic squat, which increases recruitment of the BF and EO; the isometric squat, which demands higher RF activation; the elbow plank exercise, which increases recruitment of the ES muscle; and lastly the hand plank exercise, which increases ES and EO recruitment. The greater activation suggests that performing these exercises in unstable conditions on an aquatic platform could be a training alternative, since water turbulence causes a slight increase in muscle activation.

## Figures and Tables

**Table 1:** Exercise Protocol.

Exercise	Environment	Duration	Recovery	Abbreviations
Dynamic Squat	Land	10″	40″	LE
Isometric Squat	Land	10″	40″	LE
Elbow Plank	Land	10″	40′	LE
Hand Plank	Land	10″	40″	LE
Dynamic Squat	Aquatic without turbulence	10″	40″	AE_WT
Isometric Squat	Aquatic without turbulence	10″	40″	AE_WT
Elbow Plank	Aquatic without turbulence	10″	40″	AE_WT
Hand Plank	Aquatic without turbulence	10″	40″	AE_WT
Dynamic Squat	Aquatic with turbulence from right side	10″	40″	AE_T1
Isometric Squat	Aquatic with turbulence from right side	10″	40″	AE_T1
Elbow Plank	Aquatic with turbulence from right side	10″	40″	AE_T1
Hand Plank	Aquatic with turbulence from right side	10″	40″	AE_T1
Dynamic Squat	Aquatic with turbulence from both sides	10″	40″	AE_T2
Isometric Squat	Aquatic with turbulence from both sides	10″	40″	AE_T2
Elbow Plank	Aquatic with turbulence from both sides	10″	40″	AE_T2
Hand Plank	Aquatic with turbulence from both sides	10″	40″	AE_T2

**Table 2:** Descriptive analysis (mean ± standard deviation) of EMG magnitude (%) and inferential analysis of the activation of the erector spinae muscle as a function of the exercises and taking into account the context factor.

Muscle	Exercise	Context				F	*p*-Value	η²p	Post-Hoc	Magnitude of Difference Cohen’s d [95% CI]
		LE	AE_WT	AE_T1	AE_T2					
Erector Spinae	Dynamic Squat	32.3 ± 6.7	33.7 ± 4.5	31.5 ± 6.1	33.9 ± 5.3	1.03	0.392	0.086	-	-
	Isometric Squat	30.1 ± 6.4	32.6 ± 6.2	32.6 ± 3.6	33 ± 4.2	0.92	0.441	0.077	-	-
	Elbow Plank	4.9 ± 2.1	6.2 ± 2.6	7.2 ± 3.9	7.0 ± 2.4	4.02	0.015	0.268	b, c	b: 0.60 [1.21, −0.03] c: 1.14 [1.86, 0.39]
	Hand Plank	4.3 ± 1.7	4.9 ± 1.7	5.5 ± 3.2	6.8 ± 3.1	4.49	0.009	0.290	c	c: 0.80 [1.44, 0.13]

Abbreviations: LE = Land environment; AE_WT = Aquatic environment without turbulence; AE_T1 = Aquatic environment with turbulence on one side; AE_T2 = Aquatic environment with turbulence on two sides; CI = Confidence interval differences in pairs: (a) LE vs. AE_WT; (b) LE vs. AE_T1; (c) LE vs. AE_T2; (d) AE_WT vs. AE_T1; (e) AE_WT vs. AE_T2; (f) AE_T1 vs. AE_T2.

**Table 3:** Descriptive analysis (mean ± standard deviation) of EMG magnitude (%) and inferential analysis of the activation of the rectus femoris muscle as a function of the exercises and taking into account the context factor.

Muscle	Exercise	Context				F	*p*-Value	η²p	Post-Hoc	Magnitude of Difference Cohen’s d [95% CI]
		LE	AE_WT	AE_T1	AE_T2					
Rectus Femoris	Dynamic Squat	6.4 ± 4	7.8 ± 3.6	7.7 ± 2.9	8.3 ± 3.8	2.06	0.125	0.158	-	-
	Isometric Squat	4.5 ± 2.5	5.5 ± 3.1	6.3 ± 3.3	8.3 ± 6.4	3.24	0.034	0.227	c	c: 0.625 [1.23, −0.01]
	Elbow Plank	46.9 ± 12.4	42.7 ± 6.7	41.7 ± 6.3	41.1 ± 7.2	1.36	0.271	0.110	-	-
	Hand Plank	33.3 ± 6.5	34.7 ± 6	33.7 ± 5.6	37.4 ± 6.9	1.32	0.285	0.107	-	-

Abbreviations: LE = Land environment; AE_WT = Aquatic environment without turbulence; AE_T1 = Aquatic environment with turbulence on one side; AE_T2 = Aquatic environment with turbulence on two sides; CI = Confidence interval differences in pairs: (a) LE vs. AE_WT; (b) LE vs. AE_T1; (c) LE vs. AE_T2; (d) AE_WT vs. AE_T1; (e) AE_ST vs. AE_T2; (f) AE_T1 vs. AE_T2.

**Table 4:** Descriptive analysis (mean ± standard deviation) of EMG magnitude (%) and inferential analysis of the activation of the biceps femoris muscle as a function of the exercises and taking into account the context factor.

Muscle	Exercise	Context				F	*p*-Value	η²p	Post-Hoc	Magnitude of Difference Cohen’s d [95% CI]
		LE	AE_WT	AE_T1	AE_T2					
Biceps Femoris	Dynamic Squat	5.2 ± 2.9	6.9 ± 3.7	7.8 ± 4.8	8.1 ± 5.2	4.21	0.013	0.277	b, c	b: 0.772 [1.41, 0.11] c: 0.728 [1.36, 0.07]
	Isometric Squat	4.3 ± 1.9	4.2 ± 1.9	4.2 ± 2.2	5.3 ± 3.6	1.17	0.335	0.096	-	-
	Elbow Plank	46 ± 13.7	41.7 ± 13.7	40.3 ± 11.7	39.1 ± 10.8	1.76	0.174	0.138	-	-
	Hand Plank	34.3 ± 13.3	38.9 ± 11.4	38 ± 12.2	38.1 ± 8.7	1.1	0.363	0.091	-	-

Abbreviations: LE = Land environment; AE_WT = Aquatic environment without turbulence; AE_T1 = Aquatic environment with turbulence on one side; AE_T2 = Aquatic environment with turbulence on two sides; CI = Confidence interval differences in pairs: (a) LE vs. AE_WT; (b) LE vs. AE_T1; (c) LE vs. AE_T2; (d) AE_WT vs. AE_T1; (e) AE_ST vs. AE_T2; (f) AE_T1 vs. AE_T2.

**Table 5:** Descriptive analysis (mean ± standard deviation) of EMG magnitude (%) and inferential analysis of the activation of the rectus abdominis muscle as a function of the exercises and taking into account the context factor.

Muscle	Exercise	Context				F	*p*-Value	η²p	Post-Hoc	Magnitude of Difference Cohen’s d [95% CI]
		LE	AE_WT	AE_T1	AE_T2					
Rectus Abdominis	Dynamic Squat	31.1 ± 7.4	27.6 ± 9.3	28.4 ± 6.3	27.8 ± 7.1	1.36	0.273	0.110		
	Isometric Squat	32.9 ± 7.9	31.7 ± 7.3	32.8 ± 8.4	32.4 ± 6.5	0.15	0.928	0.014		
	Elbow Plank	17.6 ± 7.6	15.4 ± 7.8	16.5 ± 8.6	16.3 ± 5.8	0.39	0.759	0.034		
	Hand Plank	15 ± 6.4	15.1 ± 7.8	17.2 ± 5.1	17.4 ± 9.1	1.97	0.137	0.152		

Abbreviations: LE = Land environment; AE_WT = Aquatic environment without turbulence; AE_T1 = Aquatic environment with turbulence on one side; AE_T2 = Aquatic environment with turbulence on two sides; CI = Confidence interval differences in pairs: (a) LE vs. AE_WT; (b) LE vs. AE_T1; (c) LE vs. AE_T2; (d) AE_WT vs. AE_T1; (e) AE_ST vs. AE_T2; (f) AE_T1 vs. AE_T2.

**Table 6:** Descriptive analysis (mean ± standard deviation) of EMG magnitude (%) and inferential analysis of the activation of the rectus oblique muscle as a function of the exercises and taking into account the context factor.

Muscle	Exercise	Context				F	*p*-Value	η²p	Post-Hoc	Magnitude of Difference Cohen’s d [95% CI]
		LE	AE_WT	AE_T1	AE_T2					
Rectus Oblique	Dynamic Squat	24.6 ± 10.2	29.2 ± 7.2	29.6 ± 6.3	32.5 ± 7.1	5.56	0.003	0.336	c	c: 0.822 [1.7, 0.15]
	Isometric Squat	17.5 ± 7.9	19.7 ± 7.2	19.8 ± 9.9	21.1 ± 8.4	1.84	0.159	0.143	-	-
	Elbow Plank	7.8 ± 3.1	7.8 ± 3.5	7.5 ± 3.4	8.3 ± 3.2	0.68	0.572	0.058	-	-
	Hand Plank	6.6 ± 2.8	7 ± 2.4	7.4 ± 2.4	8.7 ± 3.9	4.63	0.008	0.296	c, e	c: 0.831 [1.48, 0.16] e: 0.620 [1.23,–0.01]

Abbreviations: LE = Land environment; AE_WT = Aquatic environment without turbulence; AE_T1 = Aquatic environment with turbulence on one side; AE_T2 = Aquatic environment with turbulence on two sides; CI = Confidence interval differences in pairs: (a) LE vs. AE_WT; (b) LE vs. AE_T1; (c) LE vs. AE_T2; (d) AE_WT vs. AE_T1; (e) AE_ST vs. AE_T2; (f) AE_T1 vs. AE_T2.

## Data Availability

Not applicable.
